# Feasibility of risk assessment for breast cancer molecular subtypes

**DOI:** 10.1007/s10549-024-07404-9

**Published:** 2024-06-25

**Authors:** Anne Marie McCarthy, Sarah Ehsan, Kevin S. Hughes, Constance D. Lehman, Emily F. Conant, Despina Kontos, Katrina Armstrong, Jinbo Chen

**Affiliations:** 1 Department of Biostatistics, Epidemiology and Informatics, University of Pennsylvania, Blockley Hall, Room 833, 423 Guardian Drive, Philadelphia, PA 19104 USA; 2 Department of Surgery, Medical University of South Carolina, Charleston, SC 29425 USA; 3 Massachusetts General Hospital, Boston, MA USA; 4 Department of Radiology, Perelman School of Medicine, Hospital of the University of Pennsylvania, Philadelphia, PA USA; 5 Columbia University Irving Medical Center, New York, NY USA

**Keywords:** Breast cancer, Risk assessment, Tumor subtypes, Risk prediction model, Triple-negative breast cancer

## Abstract

**Purpose:**

Few breast cancer risk assessment models account for the risk profiles of different tumor subtypes. This study evaluated whether a subtype-specific approach improves discrimination.

**Methods:**

Among 3389 women who had a screening mammogram and were later diagnosed with invasive breast cancer we performed multinomial logistic regression with tumor subtype as the outcome and known breast cancer risk factors as predictors. Tumor subtypes were defined by expression of estrogen receptor (ER), progesterone receptor (PR), and human epidermal growth factor receptor 2 (HER2) based on immunohistochemistry. Discrimination was assessed with the area under the receiver operating curve (AUC). Absolute risk of each subtype was estimated by proportioning Gail absolute risk estimates by the predicted probabilities for each subtype. We then compared risk factor distributions for women in the highest deciles of risk for each subtype.

**Results:**

There were 3,073 ER/PR+ HER2 − , 340 ER/PR +HER2 + , 126 ER/PR−ER2+, and 300 triple-negative breast cancers (TNBC). Discrimination differed by subtype; ER/PR−HER2+ (AUC: 0.64, 95% CI 0.59, 0.69) and TNBC (AUC: 0.64, 95% CI 0.61, 0.68) had better discrimination than ER/PR+HER2+ (AUC: 0.61, 95% CI 0.58, 0.64). Compared to other subtypes, patients at high absolute risk of TNBC were younger, mostly Black, had no family history of breast cancer, and higher BMI. Those at high absolute risk of HER2+ cancers were younger and had lower BMI.

**Conclusion:**

Our study provides proof of concept that stratifying risk prediction for breast cancer subtypes may enable identification of patients with unique profiles conferring increased risk for tumor subtypes.

**Supplementary Information:**

The online version contains supplementary material available at 10.1007/s10549-024-07404-9.

## Introduction

Despite reductions in cancer mortality in the past several decades, breast cancer remains the second leading cause of cancer death among women in the U.S. [1]. Breast cancer has been classified into four main subtypes based on tumor molecular profiling [2]. In clinical practice, immunohistochemistry is typically used to classify tumors into subtypes defined by expression of estrogen receptor (ER), progesterone receptor (PR), and human epidermal growth factor receptor 2 (HER2). Tumors expressing ER and PR respond to endocrine therapies, and tumors expressing HER2 are treated with the anti-HER2 antibody drug Trastuzumab. Tumors that do not express ER, PR, or HER2, termed triple-negative breast cancers (TNBCs), do not respond to endocrine therapy or HER2-targeted therapy. TNBCs tend to be more aggressive, more likely to recur, and have higher mortality than other breast cancer subtypes [3, 4]. Breast cancer subtypes also display etiologic heterogeneity, with some risk factors having different or opposite associations with TNBC versus hormone receptor-positive subtypes [5]. For example, while prior biopsy and atypical hyperplasia are strongly associated with ER/PR + HER2 − breast cancers and are included in several existing breast cancer risk prediction models [6–9], there appears to be no association between these factors and TNBC [5]. We previously demonstrated that existing breast cancer risk prediction models perform more poorly in predicting TNBC compared to other subtypes [10]. Lack of accounting for different risk profiles of breast cancer subtypes may contribute to existing breast cancer risk prediction models’ limited discriminatory accuracy. Risk models developed for specific breast cancer subtypes may improve discriminatory accuracy and would be useful to direct more intensive screening to women at highest risk for cancer, particularly poor prognosis TNBC, for whom early detection may help improve outcomes. The purpose of this study was to evaluate the ability to discriminate between breast cancer subtypes using known breast cancer risk factors among a large cohort of women undergoing mammography screening in the U.S.

## Methods

We utilized a case-only design to evaluate differences in risk factors by tumor subtypes and derive the probabilities of each subtype given a breast cancer diagnosis. The absolute risk for each subtype is then obtained by proportioning the Breast Cancer Risk Assessment Tool (BCRAT) 5-years or lifetime risk according to these subtype probabilities. Methods for computing BCRAT absolute risk scores have been previously described [10].

The study population included a cohort of women ages 40–84 years old who had a screening mammogram at Massachusetts General Hospital (MGH), Newton Wellesley Hospital (NWH), or the University of Pennsylvania Health System (Penn) from 2006 to 2015 and who were diagnosed with invasive breast cancer at least 6 months after their initial mammogram. Patients completed a questionnaire at the time of the mammogram assessing reproductive history and family history. Body mass index (BMI) and breast density were determined from electronic health records (EHR). Missing BMI was supplemented with the closest available measurement from EHR within 1 year prior to or 6 months after the mammogram. Missing information on prior breast biopsy was also supplemented using EHR biopsy reports. Race/ethnicity was based on identity self-reported in EHR and categorized as Asian/Pacific Islander, Black/African American, Hispanic/Latino, White, or Other/Unknown, with the last category, including patients who reported other racial/ethnic groups or who had missing data on race/ethnicity in EHR. Breast cancer diagnoses were obtained from the Massachusetts Cancer Registry for MGH and NWH and from the state cancer registries of Pennsylvania, New Jersey, and Delaware for Penn. Patients from all three sites were also linked to each site’s institutional cancer registry. Cancer diagnoses from state cancer registries were available through 2016, while diagnoses from institutional registries were available through 2018.

For individuals with multiple mammograms over the study period, risk factors were extracted at the time of the earliest mammogram. Patients were included if they were diagnosed with invasive breast cancer at least 6 months after the mammogram, were not missing information on molecular subtype, and had not been diagnosed with breast cancer at any time prior to their initial mammogram. Individuals with breast implants, with a known *BRCA1/2* mutation or who were missing breast density information were excluded. This resulted in a final study sample of 3839 individuals with invasive breast cancer. Tumor subtypes were classified into four mutually exclusive categories based on immunohistochemistry: (1) ER or PR positive and HER2 negative (ER/PR+HER2−), (2) ER or PR positive and HER2 positive (ER/PR+HER2+), (3) ER and PR negative and HER2 positive (ER&PR−HER2+), and (4) ER and PR and HER2 negative (triple-negative breast cancer, TNBC).

Multinomial logistic regression was used to estimate associations of risk factors with the four-level outcome breast cancer molecular subtype, using ER/PR+HER2− as the reference category. Age, race, prior breast biopsy, atypical hyperplasia, age at menarche, age at first live birth, family history of breast cancer, BMI, and breast density were assessed as predictors. Atypical hyperplasia was removed from the final model due to lack of statistical significance and low prevalence in our data. Furthermore, we tested interaction terms between BMI and menopause status, BMI and breast density, and BMI and race, but these were dropped from the final model as they were not statistically significant. Calibration plots were derived by plotting the proportion of each observed outcome against the predicted probabilities within deciles. Discrimination was assessed using the area under the receiver operating curve (AUC) for each subtype. AUCs were calculated using a one vs. rest approach, in which the predicted probabilities for each subtype were classified using a dummy variable taking the value of 1 if the patient had that subtype and 0 if they had any other subtype [11]. Multiple imputation using chained equations (MICE) was used to fill in additional missing data for BMI, age at menarche, and age at first live birth. Model estimates, predictions, and AUCs were pooled across 50 imputed datasets. Absolute risk estimates were generated by multiplying the 5-years and lifetime BCRAT absolute risk estimates by the predicted probability from the new model. Patients were stratified in deciles of absolute 5-years risk and characteristics of patients in the top decile of risk for each subtype were compared. Analyses were conducted using Stata 17 (College Station, TX).

## Results

Table 1 shows the distribution of risk factors for the study population. Among 3839 invasive breast cancers, there were 3073 ER/PR+HER2− , 340 ER/PR+HER2+, 126 ER&PR−HER2+, and 300 TNBCs. Women with HER2+ disease were younger at diagnosis than both ER/PR+HER2− and TNBC and women with ER/PR−HER2+ disease were more likely to report a family history of breast cancer. A smaller proportion of women with TNBC had prior biopsies or atypical hyperplasia. Women with TNBC had earlier age at first birth and were more likely to be Black.

**Table 1:** Risk factors by invasive tumor subtype

Characteristics	Breast cancer subtype			
	ER/PR + HER2 − (*n* = 3073)	ER/PR + HER2 + (*n* = 340)	ER&PR − HER2 + (*n* = 126)	TNBC (*n* = 300)
Gail 5-years risk score, median (IQR)	1.51 (1.08, 1.95)	1.29 (0.89, 1.76)	1.29 (0.94, 1.76)	1.42 (1.02, 1.79)
Gail lifetime risk score, median (IQR)	9.01 (6.23, 12.15)	9.87 (7.39, 13.01)	10.13 (7.48, 13.59)	8.44 (6.29, 10.94)
Age at screening (years), median (IQR)	57 (48, 65.3)	53 (44, 60.7)	51.1 (45, 58.9)	55.8 (48, 64.35)
Gail 5-years risk > 1.67%, *n* (%)	1,234 (40.2%)	102 (30.0%)	37 (29.4%)	102 (34.0%)
Age (years), *n* (%)				
40–49	898 (29.2%)	137 (40.3%)	53 (42.1%)	96 (32.0%)
50–59	871 (28.3%)	105 (30.9%)	44 (34.9%)	90 (30.0%)
60–69	825 (26.8%)	71 (20.9%)	16 (12.7%)	74 (24.7%)
70–79	479 (15.6%)	27 (7.9%)	13 (10.3%)	40 (13.3%)
Number of previous biopsies, *n* (%)				
0, Unknown	2426 (78.9%)	267 (78.5%)	105 (83.3%)	253 (84.3%)
1	496 (16.1%)	49 (14.4%)	15 (11.9%)	33 (11.0%)
2 +	151 (4.9%)	24 (7.1%)	6 (4.8%)	14 (4.7%)
Atypical hyperplasia, *n* (%)				
No	3012 (98.0%)	328 (96.5%)	123 (97.6%)	299 (99.7%)
Yes	61 (2.0%)	12 (3.5%)	3 (2.4%)	1 (0.3%)
Age at menarche (years), *n* (%)				
7–11	563 (18.3%)	59 (17.4%)	26 (20.6%)	57 (19.0%)
12–13	1657 (53.9%)	185 (54.4%)	62 (49.2%)	154 (51.3%)
14	666 (21.7%)	77 (22.6%)	26 (20.6%)	63 (21.0%)
Missing/unknown	187 (6.1%)	19 (5.6%)	12 (9.5%)	26 (8.7%)
Age at first live birth (years), *n* (%)				
N/A (no births)	754 (24.5%)	85(25.0%)	24 (19.0%)	69 (23.0%)
< 20	218 (7.1%)	35 (10.3%)	6 (4.8%)	42 (14.0%)
20–24	617 (20.1%)	47 (13.8%)	28 (22.2%)	64 (21.3%)
25–29	664 (21.6%)	77 (22.6%)	29 (23.0%)	60 (20.0%)
30+	722 (23.5%)	88 (25.9%)	34 (27.0%)	54 (18.0%)
Missing/unknown	98 (3.2%)	8 (2.4%)	5 (4.0%)	11 (3.7%)
Number of first-degree relatives with breast cancer, *n* (%)				
0	2528 (82.3%)	286 (84.1%)	99 (78.6%)	255 (85.0%)
1	480 (15.6%)	49 (14.4%)	26 (20.6%)	37 (12.3%)
2	65 (2.1%)	5 (1.5%)	1 (0.8%)	8 (2.7%)
Race/ethnicity, *n* (%)				
Asian/Pacific Islander	95 (3.1%)	11 (3.2%)	6 (4.8%)	10 (3.3%)
Black/African American	275 (8.9%)	32 (9.4%)	16 (12.7%)	81 (27.0%)
Hispanic/Latino	41 (1.3%)	10 (2.9%)	2 (1.6%)	5 (1.7%)
White	2598 (84.5%)	280 (82.4%)	99 (78.6%)	202 (67.3%)
Other/Unknown	64 (2.1%)	7 (2.1%)	3 (2.4%)	2 (0.7%)
BMI (kg/m^2^), *n* (%)				
< 25	991 (32.2%)	123 (36.2%)	52 (41.3%)	83 (27.7%)
25–29	772 (25.1%)	76 (22.4%)	25 (19.8%)	88 (29.3%)
30+	733 (23.9%)	84 (24.7%)	33 (26.2%)	76 (25.3%)
Unknown	577 (18.8%)	57 (16.8%)	16 (12.7%)	53 (17.7%)
BI-rads breast density, *n* (%)				
Almost entirely fat	135 (4.4%)	14 (4.1%)	2 (1.6%)	9 (3.0%)
Scattered fibroglandular tissue	1052 (34.2%)	95 (27.9%)	37 (29.4%)	114 (38.0%)
Heterogeneously dense	1552 (50.5%)	196 (57.6%)	73 (57.9%)	152 (50.7%)
Extremely dense	277 (9.0%)	33 (9.7%)	13 (10.3%)	17 (5.7%)
Unknown	57 (1.9%)	2 (0.6%)	1 (0.8%)	8 (2.7%)

The multinomial regression model (Table 2) included age, race/ethnicity, atypical hyperplasia, number of biopsies, age at menarche, age at first live birth, first degree family history of breast cancer, BMI category, and breast density. In this model, age was associated with slightly lower likelihood of ER/PR + HER2+ , ER/PR−HER2+, and TNBC compared to the ER/PR + HER2 − eference group. Black women had a higher likelihood of ER&PR −HR2 + (RR: 1.85, 95% CI 1.04, 3.31) and TNBC (RRR: 3.64, 95% CI 2.65–5.0) compared with ER/PR+HER2 − . Prior breast biopsy was associated with a lower likelihood of TNBC (RRR: 0.71, 95% CI 0.51, 0.99).

**Table 2:** Multinomial logistic regression

Characteristics	Breast cancer subtype					
	ER/PR +HER2+		ER&PR−HER2−		TNBC	
	Median	(IQR)	Median	(IQR)	Median	(IQR)
Age at screening (years)	0.968**	(0.956, 0.979)	0.962**	(0.943, 0.980)	0.988*	(0.976, 1.000)
Race/ethnicity						
Asian/Pacific Islander vs. White	0.928	(0.487, 1.770)	1.383	(0.583, 3.280)	1.261	(0.642, 2.477)
Black/African American vs. White	1.072	(0.709, 1.620)	1.851*	(1.036, 3.307)	3.638**	(2.650, 4.995)
Hispanic/Latino vs. White	1.853	(0.897, 3.829)	1.169	(0.273, 5.012)	1.425	(0.550, 3.694)
Other/unknown vs. White	0.979	(0.440, 2.175)	1.314	(0.402, 4.300)	0.379	(0.0917, 1.563)
Previous biopsies	1.113	(0.841, 1.472)	0.757	(0.466, 1.232)	0.708*	(0.508, 0.987)
Age at menarche (years)						
12–13 vs. < 12	1.080	(0.789, 1.478)	0.820	(0.514, 1.309)	0.948	(0.685, 1.311)
14 + vs. < 12	1.070	(0.744, 1.538)	0.784	(0.447, 1.373)	0.926	(0.633, 1.353)
Age at first live birth (years)						
< 20 vs. no births	1.529	(0.977, 2.392)	0.901	(0.353, 2.302)	1.371	(0.878, 2.142)
20–29 vs. no births	0.983	(0.731, 1.322)	1.612	(0.984, 2.642)	1.066	(0.779, 1.460)
30 + vs. no births	0.923	(0.670, 1.273)	1.217	(0.709, 2.088)	0.825	(0.565, 1.206)
Family history of breast cancer	0.896	(0.657, 1.221)	1.354	(0.869, 2.108)	0.875	(0.624, 1.226)
BMI (kg/m^2^)						
25–29 vs. < 25	0.897	(0.668, 1.204)	0.669	(0.409, 1.094)	1.203	(0.872, 1.661)
30 + vs. < 25	1.027	(0.744, 1.416)	0.916	(0.554, 1.514)	0.929	(0.639, 1.350)
Dense breasts	1.144	(0.867, 1.508)	1.168	(0.748, 1.823)	1.032	(0.775, 1.373)

*p < 0.05, **p < 0.001

**Fig. 1:**
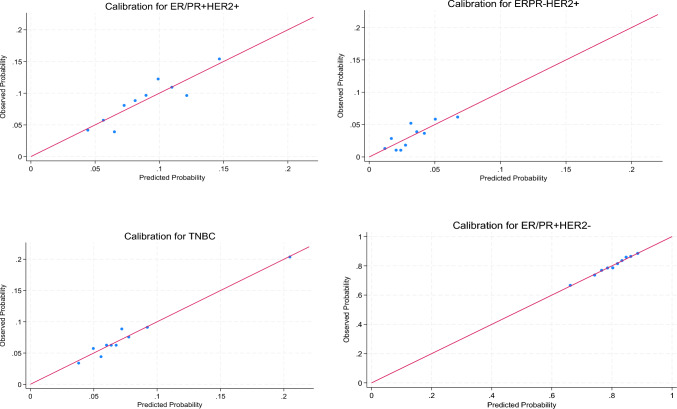
Model calibration by tumor subtypes

Model calibration was evaluated by plotting the observed versus the expected within deciles of predicted probabilities of each subtype (Fig. 1). We observed good calibration for all three subtypes compared with ER/PR+HER2 − , and calibration was particularly good for TNBC. We observed moderately good discrimination by subtype, as measured by AUCs ranging from 0.61 to 0.64 (Table 3).

**Table 3:** Model discrimination

Breast cancer subtype	AUC (95% CI)
ER/PR+HER2−	0.6096 (0.5873, 0.6320)
ER/PR+ ER2+	0.6091 (0.5790, 0.6391)
ER&PR−HER2 +	0.6430 (0.5943, 0.6917)
TNBC	0.6427 (0.6083, 0.6772)

Table 4 compares characteristics of women in the uppermost deciles of risk for each breast cancer subtype. In contrast to other subtypes, most patients at high risk of TNBC were Black, had no known family history of breast cancer, had a BMI of over 25 kg/m^2^, and were more likely to have younger age at first live birth than patients with ER/PR+HER2 − disease. Women in the highest decile of TNBC risk had lower percentage of dense breasts (57%) compared to other subtypes (76–88%). Those at high risk of HER2 + cancers were on average younger than those at high risk of other cancers, and mostly had BMIs of less than 25 kg/m^2^. The median five-year BCRAT absolute risk estimate ranged from 1.73% among patients at highest risk for TNBC to 3.45% for those at highest risk for ER/PR+ HER2− breast cancer. Absolute risk estimates generated by multiplying the 5-years and lifetime BCRAT absolute risk estimates by predicted probability from the new model are displayed for various strata of risk factors in Online Resources 1 and 2.

**Table 4:** Black and White women in the uppermost decile of absolute 5-years risk for breast cancer subtypes

Characteristics	Breast cancer subtype			
	ER/PR +HER2 −	ER/PR +HER2+	ER&PR−HER2+	TNBC
Gail 5-Years Risk score, median (IQR)	3.45 (3.09, 4.09)	3.03 (2.38, 4.00)	2.77 (2.15, 3.66)	1.73 (1.35, 3.28)
Age (years), mean +/ −SD median (IQR)	65.89 + / −8.34 66 (60.7, 72)	56.80 + / − 9.46 57 (49, 63.3)	55.64 + / − 9.02 56 (48, 62)	62.99 + / − 9.08 63 (56, 69.8)
Race/ethnicity, *n* (%)				
White	349 (97.49%)	343 (95.81%)	319 (89.11%)	86 (24.02%)
Black/African American	9 (2.51%)	15 (4.19%)	39 (10.89%)	272 (75.98%)
Prior biopsy, *n* (%)				
No	210 (58.66%)	135 (37.71%)	255 (71.23%)	286 (79.89%)
Yes	148 (41.34%)	223 (62.29%)	103 (28.77%)	72 (20.11%)
Age at menarche (years), *n* (%)				
7–11	74 (21.26%)	58 (16.57%)	96 (27.35%)	93 (27.68%)
12–13	207 (59.48%)	227 (64.86%)	195 (55.56%)	189 (56.25%)
14 +	67 (19.25%)	65 (18.57%)	60 (17.09%)	54 (16.07%)
Age at first live birth (years), *n* (%)				
N/A (No births)	82 (23.23%)	83 (23.38%)	33 (9.4%)	61 (17.84%)
< 20	14 (3.97%)	40 (11.27%)	6 (1.71%)	111 (32.46%)
20–29	182 (51.56%)	125 (35.21%)	210 (59.83%)	151 (44.15%)
30 +	75 (21.25%)	107 (30.14%)	102 (29.06%)	19 (5.56%)
Family history of breast cancer, *n* (%)				
No family history	116 (32.4%)	152 (42.46%)	50 (13.97%)	244 (68.16%)
Prior family history	242 (67.6%)	206 (57.54%)	308 (86.03%)	114 (31.84%)
BMI (kg/m^2^), *n* (%)				
< 25	112 (39.02%)	132 (46.32%)	157 (53.95%)	57 (19.39%)
25–29	96 (33.35%)	69 (24.21%)	42 (14.43%)	109 (37.07%)
30 +	79 (27.53%)	84 (29.47%)	92 (31.62%)	128 (43.54%)
Breast density, *n* (%)				
Non-dense	154 (43.87%)	85 (24.15%)	88 (25.21%)	201 (56.94%)
Dense	197 (56.13%)	267 (75.85%)	261 (74.79%)	152 (43.06%)

## Discussion

In this study we explored the feasibility of a breast cancer subtype-specific risk prediction model. Using a two-stage approach, we first used a case-only multinomial model that was then used to proportion absolute risk estimates obtained from the BCRAT model. The model, which utilized established breast cancer risk factors, showed good discrimination between breast cancer subtypes and was well calibrated. Patients with high risk for TNBC were more likely to be Black and were considerably less likely to have had a prior biopsy than patients with ER/PR+ HER2− breast cancers. Our results suggest that estimating risk for specific breast cancer subtypes, specifically TNBC, may better identify women at highest risk for this poor prognosis cancer than existing breast cancer risk prediction models.

While numerous studies have identified distinct risk factor profiles between breast cancer subtypes [5, 12], to our knowledge there are no validated breast cancer subtype-specific risk models that incorporate ER, PR, and HER2 status. Our prior work found that AUCs from the BCRAT, BCSC, BRCAPRO, and BRCAPRO + BCRAT models were all considerably lower for HER2 + and triple-negative breast cancers (AUC range 0.513–0.585) than for ER/PR+HER2 − disease (AUC range 0.605–0.629) [10]. The Rosner–Colditz breast cancer incidence model provides prediction of subtypes based on ER and PR only, with poorer performance for ER/PR − (AUC = 0.598) disease than ER/PR + (AUC = 0.629) disease [13]. Incorporating adolescent body size, vegetable intake, and breastfeeding duration into the model improved accuracy for ER/PR− (AUC = 0.630) [14], yet these factors tend not to be assessed clinically or recorded in electronic health records, making their use in clinical risk prediction models difficult. A model developed and validated among Black women in the U.S. that included age, family history, age at menarche, parity, breastfeeding, oral contraceptives use, oophorectomy, and breast biopsy achieved an AUC = 0.57 for ER − disease [15].

Our result that patients with TNBC were less likely to have had a prior biopsy than patients with hormone receptor-positive disease is consistent with prior studies demonstrating that prior biopsy and/or benign breast disease is associated with estrogen receptor-positive disease and not TNBC [5, 16–19]. This is believed to reflect different etiologic pathways between TNBC and other breast cancer subtypes [19, 20], as well as due to TNBC bearing benign appearing imaging characteristics on mammography, aggressive growth of TNBCs, and younger age at diagnosis, which may lead to lower likelihood of patients having biopsies prior to diagnosis [21]. Therefore, while presence of a prior biopsy significantly increases risk of estrogen receptor-positive disease, having had a prior biopsy is less relevant for risk of a future TNBC.

Our results provide proof of concept that a subtype-specific risk model may prove useful. We observed stark differences in characteristics of patients in the highest decile of risk for each subtype. Most strikingly, 75% of women at highest risk for TNBC were Black, compared with only 2% of women at highest risk for ER/PR+HER2 +  and only 20% of women at highest risk for TNBC had a prior breast biopsy, compared to 62% of ER/PR+HER2 + and 41% of ER/PR+HER2− patients. Furthermore, the median BCRAT 5-year risk score was only 1.73% compared with 3.45% among women at highest risk for ER/PR+HER2− This result highlights that women at high risk for TNBC are poorly identified by existing risk models. Given that TNBC has a younger age at onset than ER/PR+HER2 − disease and the fact that it is more likely to be diagnosed as an interval cancer [5] [22, 23], knowledge of elevated risk for TNBC might be used to begin screening earlier, screening more frequently, and/or incorporating supplemental screening with breast ultrasound or breast MRI.

The main limitation of our analysis is the lack of external validation of our model. Given that most invasive breast cancers are ER/PR+ HER2 −, developing and validating a subtype-specific risk model has proven challenging due to the need for very large prospective samples to achieve a reasonable number of TNBCs for model training and validation. Given this reality, the two-stage approach that leverages existing absolute risk models allows us to bypass the need for a prospective cohort, however, this approach makes it challenging to accommodate risk factors that are not present in the overall risk model. In addition, we lacked information on risk factors that have been shown to be differentially associated with triple-negative breast cancer, such as breastfeeding, oral contraceptive use, or early-life adiposity. In addition, an ER − specific polygenic risk score has been developed and validated, yet we lacked genetics data in this sample. Adding these important risk factors will be key to developing the most accurate subtype-specific risk model. Additional limitations include inability to evaluate temporal changes in characterization of subtypes by immunohistochemistry and lack of state cancer registry data in later years of the study.

In summary, our work suggests the potential utility of a subtype-specific approach to improve breast cancer risk prediction, particularly for women at elevated risk for TNBC. Given the promising findings, future studies incorporating additional risk factors and validating in large screening cohorts should commence.

## Supplementary Information

Below is the link to the electronic supplementary material.

Supplementary file1 (PDF 1,214 KB)

## Data Availability

Deidentified data underlying this article will be shared upon reasonable request to the corresponding author.
