# Supplementary material for: Feasibility of risk assessment for breast cancer molecular subtypes
Source: Breast Cancer Res Treat. 2024 Jun 25;208(1):103–10. doi: 10.1007/s10549-024-07404-9 (PMC11452472; doi:10.1007/s10549-024-07404-9)
Supplement: Supplementary file 1 — Supplementary file1 (PDF 1,214 KB) [file 10549_2024_7404_MOESM1_ESM.pdf]

Breast Cancer Research and Treatment

Feasibility of risk assessment for breast cancer molecular subtypes

Anne Marie McCarthy, ScM, PhD, Sarah Ehsan, MPH, BA, Kevin Hughes, MD, Constance Lehman, MD, PhD, Emily Conant, MD, Despina Kontos, PhD, Katrina Armstrong, MD, Jinbo Chen, PhD

Corresponding Author: Anne Marie McCarthy ScM, PhD, Department of Biostatistics, Epidemiology and Informatics, University of Pennsylvania, Philadelphia, PA, USA

(e-mail: annemcc@pennmedicine.upenn.edu)

Online Resource 1 Risk obtained by multiplying Gail 5-year risk score by predicted probabilities from model

Characteristics	Breast Cancer Subtype															
	ER/PR+HER2-				ER/PR+HER2+				ER/PR-HER2+				TNBC			
	Mean	SD	Min.	Max.	Mean	SD	Min.	Max.	Mean	SD	Min.	Max.	Mean	SD	Min.	Max.
Race/Ethnicity																
Asian/Pacific Islander	0.52	0.30	0.13	2.37	0.06	0.03	0.01	0.26	0.03	0.02	0.01	0.12	0.05	0.03	0.02	0.16
Black/African American	1.02	0.49	0.26	3.41	0.11	0.04	0.04	0.34	0.05	0.04	0.01	0.30	0.29	0.12	0.09	1.07
Hispanic/Latino	0.89	0.45	0.28	3.23	0.19	0.05	0.05	0.37	0.04	0.02	0.01	0.08	0.10	0.03	0.04	0.16
White	1.45	0.89	0.26	9.17	0.14	0.07	0.03	0.87	0.05	0.03	0.01	0.31	0.10	0.05	0.03	0.62
Other/Unknown	1.33	0.87	0.35	5.63	0.13	0.06	0.06	0.31	0.06	0.04	0.01	0.19	0.04	0.02	0.01	0.08
Family History of Breast Cancer																
No Family History	1.17	0.62	0.13	6.77	0.12	0.05	0.01	0.78	0.04	0.02	0.01	0.20	0.11	0.07	0.01	0.85
Prior Family History	2.26	1.22	0.44	9.17	0.20	0.09	0.05	0.87	0.10	0.05	0.02	0.31	0.17	0.12	0.02	1.07
Breast Density																
Non-Dense	1.47	0.85	0.25	9.17	0.12	0.06	0.02	0.66	0.04	0.03	0.01	0.26	0.14	0.10	0.02	1.07
Dense	1.29	0.86	0.13	8.83	0.14	0.07	0.01	0.87	0.05	0.04	0.01	0.31	0.11	0.07	0.01	0.85
Age (years)																
≤ 50	0.82	0.45	0.13	3.92	0.13	0.06	0.02	0.58	0.05	0.03	0.01	0.31	0.08	0.05	0.01	0.40
> 50	1.60	0.89	0.25	9.17	0.14	0.07	0.01	0.87	0.05	0.03	0.01	0.30	0.14	0.09	0.02	1.07
BMI (kg/m^2)																
< 30	1.36	0.89	0.13	9.17	0.13	0.07	0.01	0.87	0.05	0.03	0.01	0.31	0.12	0.08	0.01	1.07
≥ 30	1.37	0.79	0.14	8.07	0.14	0.06	0.04	0.55	0.05	0.03	0.01	0.30	0.13	0.09	0.02	0.74

Online Resource 2 Risk obtained by multiplying Gail lifetime risk score by predicted probabilities from model

Characteristics	Breast Cancer Subtype															
	ERPR+HER2-				ERPR+HER2+				ERPR-HER2+				TNBC			
	Mean	SD	Min.	Max.	Mean	SD	Min.	Max.	Mean	SD	Min.	Max.	Mean	SD	Min.	Max.
Race/Ethnicity																
Asian/Pacific Islander	3.40	1.99	0.64	14.94	0.43	0.30	0.03	1.64	0.24	0.19	0.01	1.15	0.36	0.20	0.05	1.26
Black/African American	4.77	1.86	1.17	14.27	0.60	0.36	0.05	2.33	0.32	0.27	0.02	1.77	1.44	0.66	0.23	4.53
Hispanic/Latino	5.66	1.94	0.89	11.89	1.48	0.84	0.06	4.48	0.31	0.22	0.01	1.11	0.69	0.24	0.09	1.26
White	8.44	4.11	0.98	37.07	0.98	0.66	0.04	5.72	0.36	0.31	0.01	2.97	0.65	0.31	0.06	2.90
Other/Unknown	7.86	3.73	2.27	23.34	0.91	0.58	0.11	3.50	0.42	0.36	0.05	2.17	0.24	0.13	0.05	0.74
Family History of Breast Cancer																
No Family History	6.92	3.21	0.64	37.07	0.85	0.58	0.03	5.72	0.29	0.22	0.01	1.88	0.67	0.40	0.05	3.28
Prior Family History	12.19	4.97	2.45	35.34	1.30	0.81	0.10	5.51	0.65	0.44	0.03	2.97	0.96	0.54	0.11	4.53
Breast Density																
Non-Dense	6.77	3.60	0.70	25.62	0.66	0.48	0.03	4.48	0.24	0.22	0.01	1.65	0.67	0.47	0.05	3.47
Dense	8.50	4.26	0.64	37.07	1.10	0.68	0.04	5.72	0.43	0.33	0.01	2.97	0.75	0.43	0.05	4.53
Age (years)																
≤ 50	9.57	3.96	1.90	32.58	1.52	0.63	0.34	5.72	0.61	0.33	0.13	2.97	0.94	0.46	0.21	4.53
> 50	7.08	3.92	0.64	37.07	0.67	0.45	0.03	5.01	0.24	0.20	0.01	1.68	0.62	0.40	0.05	3.75
BMI (kg/m^2)																
< 30	8.05	4.19	0.64	37.07	0.96	0.66	0.03	5.72	0.37	0.31	0.01	2.62	0.72	0.43	0.05	4.53
≥ 30	7.19	3.72	1.17	28.29	0.85	0.60	0.05	4.48	0.33	0.28	0.02	2.97	0.71	0.48	0.05	3.31

Online Resource 3 Black & White women in the uppermost decile of absolute lifetime risk for breast cancer subtypes

Characteristics	Breast Cancer Subtype			
	ERPR+HER2-	ERPR+HER2+	ERPR-HER2+	TNBC
Gail 5-Year Risk Score, mean +/- SD	2.946409 +/- 1.721307	2.118966 +/- 1.446901	2.063893 +/- 1.393619	2.007051 +/- 1.492537
Gail Lifetime Risk Score, mean +/- SD	21.03551 +/- 4.930239	19.79803 +/- 5.823677	19.08514 +/- 6.042968	14.09024 +/- 8.393731
Absolute Lifetime Risk, mean +/- SD	16.86127 +/- 4.006809	2.291327 +/- .6328739	1.049529 +/- .3099448	1.758429 +/- .4620897
Age (years), mean +/- SD	50.56676 +/- 8.20267	45.58873 +/- 5.760511	46.23324 +/- 6.000587	51.51145 +/- 7.847354
Race, n (%)				
Black/African American	2 (0.56%)	5 (1.41%)	31 (8.66%)	234 (65.36%)
White	356 (99.44%)	350 (98.59%)	327 (91.34%)	124 (34.64%)
Prior Biopsy, n (%)				
No	200 (55.87%)	195 (54.93%)	275 (76.82%)	296 (82.68%)
Yes	158 (44.13%)	160 (45.07%)	83 (23.18%)	62 (17.32%)
Age at Menarche (years), n (%)				
7-11	82 (23.16%)	56 (16.14%)	103 (29.26%)	87 (26.13%)
12-13	211 (59.6%)	227 (65.42%)	183 (51.99%)	193 (57.96%)
14+	61 (17.23%)	64 (18.44%)	66 (18.75%)	53 (15.92%)
Age at First Live Birth (years), n (%)				
N/A (No Births)	82 (23.16%)	70 (19.83%)	38 (10.76%)	67 (19.36%)
<20	5 (1.41%)	16 (4.53%)	4 (1.13%)	83 (23.99%)
20-29	111 (31.36%)	85 (24.08%)	147 (41.64%)	151 (43.64%)
30+	156 (44.07%)	182 (51.56%)	164 (46.46%)	45 (13.01%)
Family History of Breast Cancer, n (%)				
No Family History	107 (29.89%)	188 (52.96%)	125 (34.92%)	239 (66.76%)
Prior Family History	251 (70.11%)	167 (47.04%)	233 (65.08%)	119 (33.24%)
BMI (kg/m^2), n (%)				
<25	143 (48.64%)	181 (62.63%)	198 (64.71%)	71 (23.75%)
25-29	85 (28.91%)	50 (17.3%)	36 (11.76%)	118 (39.46%)
30+	66 (22.45%)	58 (20.07%)	72 (23.53%)	110 (36.79%)
Breast Density, n (%)				
Non-dense	84 (23.86%)	42 (12.03%)	56 (15.86%)	152 (42.82%)
Dense	268 (76.14%)	307 (87.97%)	297 (84.14%)	203 (57.18%)
